# The child and adolescent athlete: a review of three potentially serious injuries

**DOI:** 10.1186/2052-1847-6-22

**Published:** 2014-06-10

**Authors:** Dennis Caine, Laura Purcell, Nicola Maffulli

**Affiliations:** 1 Department of Kinesiology and Public Health Education, University of North Dakota, Grand Forks, ND, USA; 2 Department of Pediatrics, David Braley Sport Medicine and Rehabilitation Centre, McMaster University, Hamilton, ON, Canada; 3 Sports and Exercise Medicine, Queen Mary University of London, Barts and The London School of Medicine and Dentistry, William Harvey Research Institute, Centre for Sports and Exercise Medicine, Mile End Hospital, 275 Bancroft Road, London E1 4DG, UK

**Keywords:** Anterior Cruciate Ligament (ACL) Tear, Concussion, Physeal injury, Children and adolescents

## Abstract

The increased participation of children and adolescents in organized sports worldwide is a welcome trend given evidence of lower physical fitness and increased prevalence of overweight in this population. However, the increased sports activity of children from an early age and continued through the years of growth, against a background of their unique vulnerability to injury, gives rise to concern about the risk and severity of injury. Three types of injury–anterior cruciate ligament (ACL) injury, concussion, and physeal injury – are considered potentially serious given their frequency, potential for adverse long-term health outcomes, and escalating healthcare costs. Concussion is probably the hottest topic in sports injury currently with voracious media coverage and exploding research interest. Given the negative cognitive effects of concussion, it has the potential to have a great impact on children and adolescents during their formative years and potentially impair school achievement and, if concussion management is not managed appropriately, there can be long term negative impact on cognitive development and ability to resume sports participation. Sudden and gradual onset physeal injury is a unique injury to the pediatric population which can adversely affect growth if not managed correctly. Although data are lacking, the frequency of stress-related physeal injury appears to be increasing. If mismanaged, physeal injuries can also lead to long-term complications which could negatively affect ability to participate in sports. Management of ACL injuries is an area of controversy and if not managed appropriately, can affect long-term growth and recovery as well as the ability to participate in sports. This article considers the young athlete’s vulnerability to injury, with special reference to ACL injury, concussion, and physeal injury, and reviews current research on epidemiology, diagnosis, treatment, and prevention of these injury types. This article is intended as an overview of these injury types for medical students, healthcare professionals and researchers.

## Introduction

Participation of children and adolescents aged 5–18 years in organized sports is increasingly popular and widespread in Western countries. It is not uncommon for teens to train 20 or more hours each week at regional training centers, or for youngsters as young as six to eight to play organized sports and travel with select teams to compete against other teams of similar caliber [1]. Regular physical activity during childhood and adolescence improves overall health and fitness and reduces risk for many chronic diseases [2]. While physical activity prevents all-cause morbidity associated with a sedentary lifestyle, injuries can become a barrier to participation in physical activity [3].

The increased involvement of children and adolescents in organized sports beginning at an early age raises concern regarding risk and severity of sport injury. Inevitably, with increased participation and training come increasing numbers of sports injuries. Sports and recreational injuries are the leading cause of injury in youth in many countries [3]. Three types of youth sports injury–anterior cruciate ligament (ACL) injury, concussion, and physeal injury–are the focus of much recent media and scholarly attention given their frequency, potential for adverse long-term health outcomes, and escalating healthcare costs [4-7]. If not managed appropriately, they can also lead to long term complications which could negatively affect ability to continue to participate in exercise and sports as well as threaten general health maintenance and contribute to obesity [8]. For example, more than 50% of patients show early signs of irreversible osteoarthritis within 10 years of ACL reconstruction [9].

Concussion is probably the hottest topic in sports injury currently [5,10] with voracious media coverage and exploding research interest. Given the negative cognitive effects of concussion, it has the potential to have a great impact on children and adolescents during their formative years and potentially impair school achievement and, if concussion management is not managed appropriately, there can be long term negative impact on cognitive development and ability to resume sports participation. Sudden and gradual onset growth plate injury is a unique injury to the pediatric population which can adversely affect growth if not managed correctly [6]. Although data are lacking, overuse injury of the physis is believed to be a growing problem among young athletes [11]. If mismanaged, epiphyseal injuries can also lead to long-term complications which could negatively affect ability to participate in sports [12]. Management of ACL injuries is an area of controversy and if not managed appropriately, can affect long-term growth and recovery as well as the ability to participate in sports [13].

This article considers the vulnerability of children and youth to injury, three types of injuries sustained by pediatric athletes which have the potential for long-term negative impact if not managed appropriately – ACL injuries, concussions, and growth plate injuries – and discusses the epidemiology, diagnosis, treatment, and prevention of these injury types. This article is intended as an overview of these injury types for medical students, healthcare professionals and researchers.

## Epidemiology

Analysis of youth sport injury studies indicates that most injuries involve the knee and ankle [14]. For example, in a representative sample of 100 United States high schools involved in 9 sports during 2005–2007, ankle injury was most common (20.9%), followed by the knee (15.2%) [15]. However, the knee was the most common severely injured (more than 21 days’ time loss) location (29%), accounting for 44.6% of all surgeries [16]. The most common knee injuries were ligament tears (45.4%), contusions (15.2%), and torn menisci (8.0%) [16]. A focus on ACL injuries is important, as these injuries may increase the risk of osteoarthritis in the future [12]. Notably, among 102 Swedish female soccer players injured before the age of 20 years, the prevalence of radiographic OA was 51% compared with 8% in the uninjured knee, 12 years later [17].

Although gridiron football was associated with the highest rate of ACL injuries among high school athletes, of particular interest are the higher rates of ACL injury seen in adolescent female athletes compared to males in sports like soccer, basketball and baseball/softball [4]. For example, Rechel et al. [18] reported that U.S. high school girls sustained more than twice as many complete ligament sprains than boys.

As a result of their frequency of occurrence and potential for catastrophic injury, pediatric concussions are viewed as a public health concern [19,20]. It is estimated that as many as 3.8 million concussions occur in the US per year during competitive sports; however, as many as 50% of concussions may go unreported [10]. More than 250,000 patients aged 8–19 years presented to emergency departments in the U.S. for sport-related concussions between 2001 and 2005 [21]. Gesell et al. [22], using data from the High School Reporting Information Online (RIO), reported that concussions represented 8.9%, or almost one out of ten of all high school injuries. A Canadian emergency department (ED) study of head injuries involving five EDs in Edmonton, Alberta found that the majority of sport-related head injuries occurred to individuals less than 20 years of age (66%). It also found that 53.4% of head injuries in children 10–14 years of age were sport-related [23].

Incidence rates for U.S. high school concussions during the 2008–2010 years were estimated to be 2.5 per 10,000 Athletic-Exposures (AE’s) [24]. Football had the highest concussion rate (6.4), followed by boy’s ice hockey (5.4) and lacrosse (4.0). In gender comparable sports, girls had a higher concussion rate (1.7) than boys (1.0).

Physeal injuries account for between 15% and 30% of all emergency room skeletal injuries in children [25]. A systematic review of the case series literature on growth plate injuries revealed that 38.3% of 826 acute cases were sport-related, and among these 45 (14.2%) were associated with some degree of growth disturbance [6]. These injuries occur in a variety of sports, although gridiron football is most often reported [6].

Most cohort studies reporting on the nature and incidence of pediatric sports injuries do not specify the frequency or severity of physeal fractures. Among cohort studies which do report acute physeal injuries, a range of from 1 to 30 percent of injuries are reported as physeal fractures [6]. Tabulation of the number of injuries (n = 3762) and number of acute physeal injuries (n = 536) in these studies reveals that 14.3% of all injuries were acute physeal injuries. However, these studies report injuries as a percentage of all injuries and do not provide incidence data based on participant exposure. Thus, knowledge of the incidence rate of acute physeal injuries is lacking and awaits the results of cohort studies where exposure data is meticulously monitored.

Although incidence data are lacking, there is evidence of the existence of stress-related physeal injury affecting young athletes participating in a variety of sports including baseball (proximal humerus), basketball (distal femur/proximal tibia) climbing (phalanages), distance running (proximal tibia, first metatarsal growth plate), rugby (proximal tibia), gymnastics (clavicle, distal radius, proximal humerus), soccer (distal tibia/fibula), and tennis (proximal tibia) [6]. Most of these injuries resolved without growth complication during short-term follow-up. However, there are also reports of partial and complete epiphyseal closure in athletes representing basketball, baseball, dance, gymnastics, football, rugby and tennis [26-34]. These data are consistent with results from animal studies where prolonged intense physical training may precipitate pathological changes in the physis and, in extreme cases, produce growth disturbance [6].

## Vulnerability to injury

The young athlete may be particularly vulnerable to sport injury because of the physical and physiological processes of growth. Injury risk factors unique to the young athlete and related to the three injury types considered in the present article include growth plate vulnerability, possible differences between biological and chronological age, the adolescent growth spurt, and differential growth [35]. Young athletes might also be at increased risk of injury because of immature or underdeveloped coordination, skills, and perception [36].

### Growth plate vulnerability

Skeletally immature athletes are at risk for unique injuries not seen in adults, including growth plate fractures, apophysitis, apophyseal avulsion fractures, and greenstick fractures [35,37]. These unique injuries result from differences in the structure of growing bone compared to mature adult bone.

The differences between growing bone and mature bone include vulnerability of growth plates to shearing injury (at the epiphyseal-metaphyseal junction) resulting in growth plate fractures; vulnerability of apophyses to traction and strong muscle contractions resulting in apophysitis or avulsion injuries; and increased elasticity and resiliency of the metaphysis of long bones which, coupled with the thick periosteum typical of this age group, can result in greenstick or incomplete fractures [1,35,37,38]. Because of these differences, children and adolescents are more likely to injure bone or avulse an apophysis than to sprain a ligament or tear a muscle or tendon. However, it is also possible that the injury mechanism may be of sufficient magnitude and orientation to sprain a ligament or tear a muscle or tendon. Notably, ACL reconstruction in younger patients with significant growth remaining carries a risk of growth plate injury and growth disruption [39].

### Adolescent growth spurt

The adolescent growth spurt appears to be a time of increased risk for sports injury. Some studies of the frequency of growth plate and other sports- and recreation-related injuries indicate an increased occurrence of injury during pubescence [40-42] and a noteworthy association between peak height velocity and peak fracture rate [43]. Peak adolescent fracture incidence at the distal end of the radius coincides with a decline in size-corrected bone mineral density (BMD) in both boys and girls. Peak gains in bone area preceded peak gains in bone mineral content (BMC) in a longitudinal sample of boys and girls, supporting the theory that the dissociation between skeletal expansion and skeletal mineralization results in a period of relative bone weakness [44]. Overuse or repetitive microtrauma can strain the musculotendinous units which may also occur more frequently during growth spurts [37,45].

The results of recent research suggests that increased quadriceps strength, combined with increased knee laxity and no accompanying hamstring strength development during the adolescent growth spurt in girls, might contribute to a decrease in their knee joint stability during landing tasks. These musculoskeletal changes could potentially increase anterior cruciate ligament injury risk at a time of rapid height and lower limb growth [46].

Sensorimotor function is not fully mature by the time children reach adolescence and some mechanisms may actually regress during this period [47]. Deficits in a variety of these same sensorimotor mechanisms have been correlated with increased ACL injury risk [48-50]. Notably, three studies reported that neuromuscular control of knee motion and landing forces is significantly worse in females than in males during the transition from pre-pubertal to pubertal stages, with females showing regressions in control abilities [51-53].

### Differences between biological and chronological age

The structural, functional, and performance advantages of early-maturing boys in sports requiring size, strength, and power are well known. Children of the same chronological age may vary considerably in biological maturity status, particularly during adolescence, and individual differences in maturity status influence growth and performance during this period [54]. Bone age, which can be determined using standardized radiographs of the wrist, is one of the ways to assess biological age. Bone age does reflect the degree of maturity of the child, but it should be kept in mind that the appearance of bone can change between various ethnic groups.

Chronological age may add yet another dimension of individual variation, as most youth sports are categorized by chronological age. The fear is that an unbalanced competition between early- and late-maturing and/or older and younger boys in contact sports such as football and wrestling contributes to at least some of the serious injuries in these sports. For example, in a study of injury incidence in elite French youth football players, late-maturing boys sustained a significantly greater incidence rate of major injuries than early-maturing boys [55]. There were also differences between maturity groups when patterns of injury location, type, severity and re-injury were analyzed [55].

### Differential growth

The normal growth pattern is nonlinear: differential growth of the body segments (head, trunk, and lower extremities) occurs throughout growth and influences body proportions accordingly [54]. At birth, the relative contribution of head and trunk to total stature is highest, and this declines through childhood into adolescence. Thus, the child is characterized by a proportionately larger head and trunk, and shorter legs compared with an adult. This "top-heavy" characteristic could predispose the young athlete to increased risk of injuries [35]. Although data are lacking it seems logical to presume, for example, that a young ‘top-heavy’ child would be at increased risk of falling in sports which involve riding on top of animals such as sheep (mutton-busting), camels (camel-racing), horses, or on top of various vehicles, including bicycles; or at increased risk of overuse injury in sport activities involving substantial running activity.

Child and adolescent athletes may have a more prolonged recovery and are more susceptible to concussion accompanied by catastrophic injury [5,10]. Children’s greater head-to-body ratio and weaker neck muscles, combined with their relative nervous system immaturity, lesser myelinization, and thinner frontal and temporal bones, may predispose them to increased risk of head injury and concussion [56]. Concussion in the young athlete is also of specific concern because of their continuing cognitive maturation. Whereas the adult brain has achieved its operational skills for everyday life, the child’s brain is still developing in areas of concentration, establishing memory patterns, reasoning, problem-solving, and other cognitive skills [56].

## Diagnosis and management

### ACL Tear

A focus on ACL injuries is important, as these injuries may increase the risk of osteoarthritis in the future [12]. The most common mechanism of ACL injury is a non-contact pivoting motion on a fixed foot or a trauma with the knee in hyperextension [57]. If a hemarthrosis develops within a few hours after the trauma in the absence of a bony injury there is a 70% chance of ACL injury [58]. The examiner should assess gait and alignment, range of motion, and assess the affected joint and compare it with the contralateral joint, taking into account that most children may have hyperlaxity which decreases with maturity [59]. Radiographs should be examined for bony injuries. Magnetic resonance imaging (MRI) can be useful in very experienced hands [60], but may be no better than accurate clinical examination [60].

The management of ACL deficiency in skeletally mature children is still controversial, especially in terms of operative timing and surgical technique [61]. Conservative management is not recommended, as it is accompanied by marked reduction in activity, decline in functional performance, and development of early osteoarthritis [62,63]. Historically, delayed anatomic ACL reconstructions were preferred [64], recommending extensive rehabilitation and return to activities with a brace to skeletal maturity and growth plate closure, to allow an anatomical adult-like reconstruction [65]. The present trend favours early reconstruction, using either extra physeal techniques in very young athletes, or anatomical reconstruction technique placing the tibial and femoral tunnels close to the centre on the growth plate of the tibia and femur in young athletes closer to skeletal maturity.

More anatomic physeal-sparing reconstruction techniques seems to be promising, but these techniques are technically challenging. Partial transphyseal techniques avoid the distal lateral femoral physis, providing more isometric tibial graft positioning and over the top reconstructions, provide excellent stability and return to sporting activities. Complete transphyseal ACL reconstruction is very similar to adult ACL reconstructions [66]. This procedure allows ideal tunnel placement and improves graft longevity and knee function, but the incidence of growth disturbance may increase, especially in very skeletally immature children [67-70].

In Tanner stage III or IV patients receiving transphyseal quadrupled hamstring autogenous ACL reconstruction, graft fixation to the femur with a suspension device and tibial fixation with interference screw have been promising [69]. Transphyseal reconstruction is recommended for patients with Tanner II and III stages, but the evidence in Tanner stage I patients is insufficient [70].

In general, Tanner stage IV-V children are considered adolescents without substantial growth remaining, and can be treated like adults. Tanner stage III children are considered "adolescents with substantial growth remaining" and a modified transphyseal reconstruction with soft tissue graft (hamstrings) small tunnels and avoiding fixatioin across the physis can be undertaken with little risk of physeal injury. Tanner stage I and II patients have significant growth remaining. In these children, management options are (1) brace and activity followed by delayed reconstruction when older; (2) extra-physeal reconstruction; or (3) partial/complete transphyseal, but this carries the risk of significant growth arrest. Complications of ACL reconstruction are rare, and most of the documented growth complications are secondary to surgeon errors such as placement of a fixation device across a growth plate [71,72]. With careful attention to surgical technique, paediatric ACL reconstruction can be safe and effective.

### Concussion

Any direct blow to the head/face or a blow to the body that transmits a force to the brain can cause a concussion [5]. Signs and symptoms of concussion can be subtle and easily overlooked. These may include headache, nausea, dizziness, difficulty concentrating or remembering, confusion and emotional lability. Younger children may present with even more subtle signs, such as abdominal pain or behavioral changes [73]. Symptoms typically last for 7–10 days [5], although they may be prolonged for weeks to months [74-76]. In younger children, recovery may take longer [77,78]. Cognitive sequelae of concussion, including impaired memory, poor attention and lack of concentration, may negatively impact on a child’s ability to learn and attend to schoolwork [74,75,78,79].

Management of concussions in pediatric athletes generally adheres to adult guidelines outlined in the Zurich Consensus Statement [5], but should be more cautious [80,81]. Children and adolescents take longer than adults to recover after a concussion, which underscores the need for a more conservative approach to management and return to play [56]. Any child or adolescent suspected to have sustained a concussion should be immediately removed from play and not allowed to return until cleared by a physician [5,80,81]. A concussion can be evaluated on the sideline by a coach or athletic trainer using a concussion tool such as the SCAT3 or Child SCAT 3 [5,80,81]. Assessment should include a neurological exam and assessment of attention and memory.

A physician should evaluate any athlete who has sustained a concussion as soon as possible after the injury to ensure proper diagnosis. Diagnostic imaging, such as CT or MRI, is generally not required.

Studies of concussion management in paediatric patients are sparse. One study of high school and college student athletes found that cognitive and physical rest immediately after injury, as well as later during recovery, resulted in improved symptoms and increased performance on computerized neuropsychological tests [82].

Consensus agreement is that rest, both physical and mental, is the keystone of concussion management [5,80]. Physical activities, including sports and exercise, and mental activities, including video games, TV, computer work, and reading, should be limited to allow symptoms to improve. Mental rest may require that a concussed athlete abstain from school or modify assignments/tests for a period of time to allow symptoms to decrease [5,81]. As symptoms improve, students can gradually increase cognitive tasks and social activities, including school, as long as symptoms are not exacerbated [5,83].

Return to learn is a vital component of concussion management in children and adolescents [84-86]. Mental rest can be challenging for students. Recovery may be prolonged if students participate in cognitive tasks that exacerbate symptoms, known as "cognitive overexertion" [84]. Students may need to abstain from school for a day or two until symptoms improve, and then gradually return (e.g., attending half-days or only certain courses), until they are able to attend full-time without exacerbating symptoms [75,81,84-86].

Students do not need to be symptom-free to return to school. However, students may require accommodation or modifications to their schedule to allow school participation without worsening symptoms [75,81,83,84,86]. Academic accommodations/modifications may include taking frequent breaks during the day, having a quiet area they can go to; shortened assignments, more time to complete assignments; limiting tests/exams to one per day, etc. [75,80,81,83,84,86]. Full return to academics must precede return to sports. If a prolonged absence from school (more than a couple of weeks) is necessary due to persistent symptoms, referral to a specialist with expertise in concussion, as well as a neuropsychologist, may be required.

Return to play decisions for pediatric athletes following a concussion can be difficult. Because of the different physiological response and longer recovery after concussion during childhood and adolescence, a more conservative return to play approach is recommended [5,80]. No athlete should return to sport/activity until all symptoms have resolved and medical clearance has been obtained. Pediatric athletes should be symptom-free for several days [80] prior to starting a gradual return to activity following a stepwise exertion protocol [5]. Each step should take a minimum of 24 hours [5,80,87]. If any symptoms return, the athlete should rest until symptoms resolve and then try going back to the previous asymptomatic step and be reassessed by a physician.

Specific factors may require modification of concussion management [5]. These modifying factors may include medications; a history of multiple prior concussions; younger age; and co-morbid conditions such as mental illness, attention deficit hyperactivity disorder, headache disorder, and learning disabilities. The presence of modifying factors may predict the potential for prolonged recovery and require additional management considerations, including formal neuropsychological testing and diagnostic imaging [5].

Governments are becoming increasingly cognizant of the importance of concussion awareness and are taking steps to improve concussion education. In the United States, the Lystedt Law was passed in 2009 recommending concussion education for athletes, parents and coaches [88]. In Canada, the Ontario Ministry of Education has mandated that all school boards in the province develop and enforce concussion policies [89].

### Physeal injury

Disturbed physeal growth as a result of acute growth plate injury can result in limb length discrepancy, angular deformity, or altered joint mechanics [90]. Osteoarthritis may result from chondral damage at the time of growth plate injury, articular incongruity, or joint malalignment [91,92].

Epiphyseal injury may present with persistent or severe pain, visible deformity, or an inability to move or put pressure on a limb [93,94]. Swelling near a joint with focal tenderness over the physis may also be present. Lower extremity injuries may present as an inability to bear weight on the injured side; upper extremity injuries present with complaints of impaired function and reduced range of motion [95]. X-rays are typically used to determine whether a growth plate fracture has occurred. However, other diagnostic tests such as magnetic resonance imaging (MRI) or ultrasound, are also useful [96,97].

Management of acute epiphyseal plate injury depends on type of fracture. The system most widely used to describe acute growth plate injuries was developed by Salter and Harris (SH) and includes five types of injury [98]. In minimally displaced SH I and II injuries only symptomatic treatment may be necessary. However, if there is a wide displacement manipulation under anaesthesia with immobilization is indicated. The child is instructed to limit activities that impose pressure on the injured area. These injury types may be associated with growth impairment [99]. SH III and IV injuries are intraarticular, and operative anatomical reduction most often with internal fixation is necessary depending on patient age, fracture location, intra-articular displacement, and angulation. In these instances, the child needs to be followed up to skeletal maturity. Sometimes a growth arrest line may appear as a marker of the injury. SH V physeal injuries often result in partial or complete growth arrest. As a result, physeal bar resection may be required or other surgical procedures may be necessary to prevent or correct deformity [100].

Young athletes are also vulnerable to stress-related physeal injuries [6]. Symptoms of chronic epiphyseal plate injuries include pain on weight-bearing and decreased function. These injuries may not show evidence of abnormalities during early radiographs; however, growth arrest and/or angular malalignment may follow.

Physeal stress injuries are thought to develop when repetitive loading of the extremity disrupts metaphyseal perfusion which in turn inhibits ossification of the chondrocytes in the zone of provisional calcification [101]. The hypertrophic zone continues to widen as the chondrocytes continue to transition from the germinal layer to the proliferative zone [102]. Widening of the physis may be seen radiographically, whereas physeal cartilage extension into the metaphysis has been shown with magnetic resonance imaging [102,103].

Treatment for physeal stress injury is straightforward: rest from loading of the extremity [6,11,101]. However, in cases involving growth disturbance, corrective surgery may be required [29,30].

Physeal injury may also arise from ACL surgery. The current literature now supports the trend toward early operative treatment of ACL injury to restore knee stability and prevent progressive meniscal and/or articular cartilage damage, but the optimal approach to ACL reconstruction in this age group remains controversial [104]. Despite the reported clinical success of transphyseal reconstruction, iatrogenic growth disturbance secondary to physeal damage remains a genuine concern [104].

## Injury prevention

ACL injury, concussion, and growth plate injury may cause significant discomfort and disability. Unfortunately, these injuries may also result temporarily or even permanently in reduced levels of physical activity, thus negating the potential benefits of sports participation for children and adolescents. Although it is impossible to eliminate these injuries, attempts to reduce them are obviously warranted. Unfortunately, the level of evidence regarding the prevention of these injuries is quite variable. Whereas, there has been considerable research to test preventive measures for ACL injury, there is a dearth of data available related to the prevention of concussion and physeal injury.

### ACL injury

Preliminary data reveal that integrative neuromuscular training protocols implemented in pre-adolescent and early adolescent stages may artificially induce the neuromuscular spurt [105,106] and have the potential to reduce the risk of sports-related injury in young athlete [105-107]. Noyes & Barber-Westin [108] conducted a systematic review of studies which attempted to prevent ACL injuries in female athletes under the age of 19 years. Only 8 studies met inclusion criteria and of these, only three ACL intervention programs (Sportsmetrics, Prevent Injury and Enhance Performance, and Knee Injury Prevention) successfully reduced noncontact ACL injury incidence rates in female adolescent athletes. Ladenhauf et al. [109] recommend that young athletes should be encouraged to partake in preseason training programs focused on strengthening, neuromuscular and proprioceptive training units under the appropriate supervision of qualified personnel [109].

### Concussion

Surprisingly, little research has tested interventions specifically related to concussion prevention. Modification of sporting rules, including padding of soccer posts and banning of spearing in American football have reduced concussive injuries [73]. There is also evidence that helmet use reduces head injury risk in skiing, snowboarding and bicycling, but the effect on concussion risk is inconclusive [110]. It is important to recognize that helmets are not concussion-proof [80,81].

Elimination of checking in youth hockey has also been shown to reduce the frequency of concussions [111]. For example, comparison of hockey leagues in Canada for 11–12 year old players finds that compared with leagues that do not allow body checking, those that do have an associated 3-fold increased risk of game-related injuries, including severe injuries and severe concussions [111].

Research indicates knowledge regarding concussion among youth sports coaches and athletes is limited [112,113], and that nearly half of concussions that occur each year go unreported [10]. Education programs designed to increase awareness of concussive symptoms are believed to reduce the incidence and reoccurrence of concussions, but have not been tested [56]. It is believed that concussion education initiatives should focus on improving attitudes and beliefs among athletes, coaches and parents to promote better care-seeking behaviors among young athletes [114]. Notably, three years after the passage of a concussion law in Washington State in the United States, high school football and soccer coaches are receiving substantial concussion education and have good concussion knowledge [88].

Coaches, athletic trainers, parents and athletes and anyone involved in youth sports should be educated about concussion and how to recognize and manage concussions. Encouraging fair play, respect for opponents and eliminating violence can help reduce the incidence of concussions. In addition, advising children to participate in non-contact sports, such as volleyball and swimming, or in non-contact leagues may also reduce concussions [80].

### Physeal injury

Given the frequency of growth plate injury it is also surprising that little research has tested interventions specifically related to physeal injury prevention, including both acute and overuse injury. However, several preventive measures are worthy of consideration [6]. First, given the elevated susceptibility of the growth plate to injury during pubescence, coaches should reduce training loads and delay skill progressions for young athletes experiencing periods of rapid growth. Second, coaches should utilize a variety of drills or activities during practice to avoid excessively repetitive movements that may result in physeal overuse injury. Emphasis should be on quality and individualization of workouts rather than training volume. There should also be avoidance of single sports and year round sports prior to skeletal maturity, multigame tournaments, and mandatory months off for recovery. Finally, clinicians need to educate parents and coaches as to the existence of overuse physeal injury and the need for rest to ensure proper recovery and return to sport participation.

## Conclusion

The increased organized sports involvement of children from an early age, against a background of their unique characteristics and vulnerability to injury, raises concern about the risk and severity of sports-related injury in this population. Three types of injury–ACL tear, concussion, and physeal injury (both acute and overuse)–have become the focus of much recent attention due to their frequency, potential for adverse long-term health outcomes, and the financial burden of medical care. This paper provides an up-to-date review of current concepts and developments related to the epidemiology, diagnosis, management and prevention of these injuries.

## Competing interests

The authors declare that they have no competing interests.

## Authors’ contributions

All three authors made substantive contributions to this review, including feedback on sections developed by the other co-authors. All authors contributed equally to the section on ‘Vulnerability to Injury’ and ‘Epidemiology of Injury’. DC wrote the subsections related to ‘Growth Plate Injury’ as well as developing the first draft of the ‘Introduction’ and ‘Conclusion’ sections. LP wrote the subsections related to ‘Concussion’. NM wrote the subsections related to ‘ACL Tear’. All authors read and approved the final manuscript.

## Pre-publication history

The pre-publication history for this paper can be accessed here:

http://www.biomedcentral.com/2052-1847/6/22/prepub

## References

[B1] Maffulli N Caine D Maffuli N, Caine D The epidemiology of children’s team sports injuries Epidemiology of Pediatric Sports Injuries: Team Sports Med Sport Sci, Volume 49 2005 Basel: Karger 1 8 10.1159/000085330 16247259

[B2] U.S. Department of Health and Human Services Physical Activity Guidelines Advisory Committee Report 2008 Washington, DC: U.S. Department of Health and Human Services

[B3] Emery CA Injury prevention in paediatric sport-related injuries: A scientific approach Br J Sports Med 2010 44 64 69 10.1136/bjsm.2009.068353 19945972

[B4] Shea KG Grimm NL Ewing CK Aoki SK Youth sports anterior cruciate ligament and knee injury epidemiology: Who is getting injured? In what sports? When? Clin Sports Med 2011 30 691 706 10.1016/j.csm.2011.07.004 22018311

[B5] McCrory P Meewisse WH Aubry M Cantu B Dvorak J Echemendia RJ Engebretsen L Johnston K Kutcher JS Raftery M Sills A Benson B Davis GA Ellenbogen RG Guskiewicz K Herring ST Iverson GL Jordan BD Kissick J McCrea M McIntosh AS Maddocks D Makdissi M Purcell L Putukian M Schneider K Tator CH Turner M Concensus statement on concussion in sport: the 4^th^ International Conference on Concussion in Sport held in Zurich, November 2012 Br J Sports Med 2013 47 250 258 10.1136/bjsports-2013-092313 23479479

[B6] Caine D DiFiori J Maffulli N Physeal injuries in children’s and youth sports: Reasons for concern? Br J Sports Med 2006 40 749 760 10.1136/bjsm.2005.017822 16807307 PMC2564388

[B7] Nierenberg C Knee Injuries on the Rise in Young Athletes. WebMD Health News, October 17, 2011 http://www.webmd.com/children/news/20111017/knee-injuries-on-the-rise-in-young-athletes

[B8] Schub D Saluan P Anterior cruciate ligament injuries in the young athlete: evaluation and treatment Sports Med Arthrosc 2011 19 34 43 10.1097/JSA.0b013e31820b960d 21293236

[B9] Hewett TE Johnson DL ACL prevention programs: fact or fiction Orthop 2010 33 36 39 10.3928/01477447-20091124-19 20052952

[B10] Harmon KG Drezner J Gammons M Guskiewicz K Halstead M Herring S Kutcher J Pana A Putukian M Roberts W American Medical Society for Sports Medicine Position Statement: concussion in sport Clin J Sport Med 2013 23 1 18 10.1097/JSM.0b013e31827f5f93 23269325

[B11] DiFiori JP Overuse injury of the physis: a "growing" problem Clin J Sport Med 2010 20 336 337 10.1097/JSM.0b013e3181ebb55d 20818188

[B12] Caine DJ Golightly YM Osteoarthritis as an outcome of paediatric sport: an epidemiological perspective Br J Sports Med 2011 45 52 56 10.1136/bjsm.2010.081984 21330645

[B13] Frank JS Gambacorta PL Anterior cruciate ligament injuries in the skeletally immature athlete: diagnosis and management J Am Acad Orthop Surg 2013 21 78 87 10.5435/JAAOS-21-02-78 23378371

[B14] Caine D Caine C Maffulli N Incidence and distribution of pediatric sport-related injuries Clin J Sport Med 2006 16 501 514 10.1097/01.jsm.0000251181.36582.a0 17119363

[B15] Ingram JG Fields SK Yard EE Epidemiology of knee injuries among boys and girls in US High School Athletes Am J Sports Med 2008 36 1116 1122 10.1177/0363546508314400 18375784

[B16] Darrow CJ Collins CL Yard EE Comstock RD Epidemiology of severe injures among United States high school athletes, 2005–2007 Am J Sports Med 2009 37 1798 1805 10.1177/0363546509333015 19531659

[B17] Lohmander LS Ostenberg A Englund M Roos H High prevalence of knee osteoarthritis, pain, and functional limitations in female soccer players twelvey ears after anterior cruciate ligament injury Arthritis Rheum 2004 50 3145 3152 10.1002/art.20589 15476248

[B18] Rechel JA Collins CL Comstock RD Epidemiology of injuries requiring surgery among high school athletes in the United States, 2005 to 2010 J Trauma 2011 71 982 989 10.1097/TA.0b013e318230e716 21986739

[B19] Wiebe DJ Comstock RD Nance ML Concussion research: a public health priority Inj Prev 2011 17 69 70 10.1136/ip.2010.031211 21212444

[B20] MeehanWP III High school concussions in the 2008–2009 academic year Am J Sports Med 2010 38 2405 2409 10.1177/0363546510376737 20716683 PMC3120225

[B21] Bakhos LL Lockhart GR Myers R Linakis JG Emergency department visits for concussion in young child athletes Pediatrics 2010 126 e550 e556 10.1542/peds.2009-3101 20805145

[B22] Gessel LM Fields SK Collins CL Dick RW Comstock RD Concussions among United States high school and collegiate athletes J Athl Tr 2007 42 495 503 PMC2140075 18174937

[B23] Kelly KD Liseeel HL Rowe BH Vincenten JA Voaklander DC Sport and recreation-related head injuries treated in the emergency department Clin J Sport Med 2001 11 77 81 10.1097/00042752-200104000-00003 11403118

[B24] Marar M McIlvain NM Fields SK Comstock SD Incidence rates for U.S. high school concussions during the 2008–2010 academic years Am J Sports Med 2012 40 747 755 10.1177/0363546511435626 22287642

[B25] Perron AD Miller MD Brady WJ Orthopedic pitfalls in the ED: pediatric growth plate injuries Am J Emerg Med 2002 20 50 54 10.1053/ajem.2002.30096 11781914

[B26] Albanese SA Palmer AK Kerr DR Carpenter CN Lisi D Levinsohn EM Wrist pain and distal growth plate closure of the radius in gymnasts J Ped Orthop 1989 9 23 28 10.1097/01241398-198901000-00005 2915034

[B27] Bak K Boeckstyns M Epiphysiodesis for bilateral irregular closure of the distal radial physis in a gymnast Scand J Med Sci Sports 1997 7 363 366 9458504 10.1111/j.1600-0838.1997.tb00168.x

[B28] Ejnisman B Andreoli CV Pochini ADC Monteiro GC Faloppa F Cohen M Skaf AY Proximal humeral epiphysiolysis in a gymnast Acta Ortop Bras 2007 15 290 291 10.1590/S1413-78522007000500012

[B29] Howe WB Caine D Bergman GD Ross W Wrist pain-gymnastics Med Sc Sports Exerc 1997 29 S151

[B30] Laor T Wall EJ Vu LP Physeal widening in the knee due to stress injury in child athletes Am J Roentgenol 2006 186 1260 1264 10.2214/AJR.04.1606 16632716

[B31] Nanni M Butt S Mansour R Cassar-Pullicino VN Roberts A Stress-induced Salter–Harris I growth plate injury of the proximal tibia: first report Skeletal Radiol 2005 34 405 410 10.1007/s00256-004-0892-5 15782342

[B32] Sato T Shinozaki T Fukudo T Watanabe H Aoki J Yanagawa T Takagishi K Atypical growth plate closure: a possible chronic Salter and Harris Type V injury J Pediatr Orthop 2002 11 155 158 10.1097/00009957-200204000-00013 11943991

[B33] Shih C Chang CY Penn IW Chronically stressed wrists in adolescent gymnasts: MR imaging appearance Radiol 1995 195 855 859 10.1148/radiology.195.3.7754021 7754021

[B34] Shybut TB Rose DJ Strongwater AM Second metatarsal physeal arrest in an adolescent flamenco dancer: a case report Foot Ankle Int 2008 29 859 862 10.3113/FAI.2008.0859 18752788

[B35] Maffulli N Caine D Brukner P, Khan K The Younger Athlete Clinical Sports Medicine 2012 4 McGraw-Hill: Sydney

[B36] National Center for Injury Prevention and Control CDC Injury Research Agenda 2009–2018 Atlanta (GA): Centers for Disease Control and Prevention http://www.cdc.gov/injury/researchagenda/

[B37] Frank JB Jarit GJ Bravman JT Rosen JE Lower extremity injuries in the skeletally immature athlete J Am Acad Orthop Surg 2007 15 356 366 17548885 10.5435/00124635-200706000-00005

[B38] Demorest RA Landry GL Training issues in elite young athletes Curr Sport Med Reports 2004 3 167 172 10.1249/00149619-200406000-00013 15122987

[B39] AlHardy SW Anterior cruciate ligament injuries in growing skeleton Int J Health Sci 2010 4 71 79 PMC3068805 21475528

[B40] Flaschsmann R Broom ND Hardy AE Moltschaniwyskyi G Why is the adolescent joint particularly susceptible to osteochondral shear fracture? Clin Orthop Relat Res 2000 381 212 221 11127658 10.1097/00003086-200012000-00025

[B41] Schuch T Hanson C Goodwin BJ Romanick M Caine D A hospital-based study of pediatric sport and recreational injuries Med Sci Sports Exerc 2012 44 S629

[B42] Alexander CJ Effect of growth rate on the strength of the growth plate-shaft function Skeletal Radiol 1976 1 67 76 10.1007/BF00347411

[B43] Bailey DA Wedge JH McCulloch RG Martin AD Bernhardson SC Epidemiology of fractures of the distal end of the radius in children as associated with growth J Bone Joint Surg Am 1989 71 1225 1231 2777851

[B44] Faulkner RA Davison KS Bailey DA Mirwald RL Baxter-Jones AD Size-corrected BMD decreases during peak linear growth: implications for fracture incidence during adolescence J Bone Mineral Res 2006 21 1864 1870 10.1359/jbmr.060907 17002589

[B45] Brenner JS Overuse injuries, overtraining and burnout in child and adolescent athletes Pediatr 2007 119 1242 1245 10.1542/peds.2007-0887 17545398

[B46] Wild CY Steele JR Munro BJ Musculoskeletal and estrogen changes during the adolescent growth spurt in girls Med Sci Sports Exerc 2013 45 138 145 10.1249/MSS.0b013e31826a507e 22843105

[B47] Quatman-Yates CC Quatman CE Meszaros AJ Paterno MV Hewett TE A systematic review of sensorimotor function during adolescence: a developmental state of increased motor awkwardness? Brit J Sports Med 2012 46 649 655 10.1136/bjsm.2010.079616 21459874 PMC4157222

[B48] Paterno MV Schmitt LC Ford KR Rauh MJ Myer GD Huang B Hewett TE Biomechanical measures during landing and postural stability predict second anterior cruciate ligament injury after anterior cruciate ligament reconstruction and return to sport Am J Sports Med 2010 38 1968 1978 10.1177/0363546510376053 20702858 PMC4920967

[B49] Hewett TE Myer GD Ford KR Heidt RS Jr Colosimo AJ McLean SG van den Bogert AJ Paterno MV Succop P Biomechanical measures of neuromuscular control and valgus loading of the knee predict anterior cruciate ligament injury risk in female athletes: a prospective study Am J Sports Med 2005 33 492 501 10.1177/0363546504269591 15722287

[B50] Swanik CB Covassin T Stearne DJ Schatz P The relationship between neurocognitive function and noncontact anterior cruciate ligament injuries Am J Sports Med 2007 35 943 948 10.1177/0363546507299532 17369562

[B51] Ford KR Myer GD Hewett TE Longitudinal effects of maturation on lower extremity joint stiffness in adolescent athletes Am J Sports Med 2010 38 1829 1837 10.1177/0363546510367425 20522830 PMC3968426

[B52] Hewett TE Myer GD Ford KR Decrease in neuromuscular control about the knee with maturation in female athletes J Bone Jt Surg Am 2004 86-A 1601 1608 10.2106/00004623-200408000-00001 15292405

[B53] Quatman CE Ford KR Myer GD Hewett TE Maturation leads to gender differences in landing force and vertical jump performance: a longitudinal study Am J Sports Med 2006 34 806 813 16382009 10.1177/0363546505281916

[B54] Malina RM Bouchard C Bar-Or O Growth, maturation and physical activity 2004 2 Champaign: Human Kinetics

[B55] Le Gall F Carling C Reilly T Biological maturity and injury in elite youth football Scand J Med Sci Sports 2007 17 564 572 17076832 10.1111/j.1600-0838.2006.00594.x

[B56] Guskiewicz KW Valovich McLeod TC Pediatric sports-related concussion PM&R 2011 3 353 364 21497322 10.1016/j.pmrj.2010.12.006

[B57] Boden BP Dean GS Feagin JA Jr Garrett WE Jr Mechanisms of anterior cruciate ligament injury Orthopedics 2000 23 573 578 10875418 10.3928/0147-7447-20000601-15

[B58] Luhmann SJ Acute traumatic knee effusions in children and adolescents J Pediatr Orthop 2003 23 199 202 12604951

[B59] Hinton RY Rivera VR Pautz MJ Sponseller PD Ligamentous laxity of the knee during childhood and adolescence J Pediatr Orthop 2008 28 184 187 10.1097/BPO.0b013e3181652120 18388713

[B60] Kocabey Y Tetik O Isbell WM Atay OA Johnson DL The value of clinical examination versus magnetic resonance imaging in the diagnosis of meniscal tears and anterior cruciate ligament rupture Arthroscopy 2004 20 696 700 10.1016/j.arthro.2004.06.008 15346110

[B61] Maffulli N Del Buono A Anterior ligament tears in children Surgeon 2013 11 59 62 10.1016/j.surge.2012.02.003 22494779

[B62] Aichroth PM Patel DV Zorrilla P The natural history and treatment of rupture of the anterior cruciate ligament in children and adolescents. A prospective review J Bone Joint Surg (Br) 2002 84 38 41 10.1302/0301-620X.84B1.11773 11837830

[B63] Pressman AE Letts RM Jarvis JG Anterior cruciate ligament tears in children: an analysis of operative versus nonoperative treatment J Pediatr Orthop 1997 17 505 511 9364393

[B64] Woods GW O’Connor DP Delayed anterior cruciate ligament reconstruction in adolescents with open physes Am J Sports Med 2004 32 201 210 10.1177/0363546503258868 14754745

[B65] Moksnes H Engebretsen L Risberg MA Performance-based functional outcome for children 12 years or younger following anterior cruciate ligament injury: a two to nine-year follow-up study Knee Surg Sports Traumatol Arthrosc 2008 16 214 223 10.1007/s00167-007-0469-7 18157486

[B66] Andrews M Noyes FR Barber-Westin SD Anterior cruciate ligament allograft reconstruction in the skeletally immature athlete Am J Sports Med 1994 22 48 54 10.1177/036354659402200109 8129110

[B67] Cohen M Ferretti M Quarteiro M Marcondes FB de Hollanda JP Amaro JT Abdalla RJ Transphyseal anterior cruciate ligament reconstruction in patients with open physes Arthroscopy 2009 25 831 838 10.1016/j.arthro.2009.01.015 19664501

[B68] Courvoisier A Grimaldi M Plaweski S Good surgical outcome of transphyseal ACL reconstruction in skeletally immature patients using four-strand hamstring graft Knee Surg Sports Traumatol Arthrosc 2011 19 588 591 Epub 2010 Oct 2 10.1007/s00167-010-1282-2 20890694

[B69] Kocher MS Smith JT Zoric BJ Lee B Micheli LJ Transphyseal anterior cruciate ligament reconstruction in skeletally immature pubescent adolescents J Bone Joint Surg Am 2007 89 2632 2639 10.2106/JBJS.F.01560 18056495

[B70] Kaeding CC Flanigan D Donaldson C Surgical techniques and outcomes after anterior cruciate ligament reconstruction in preadolescent patients Arthroscopy 2010 26 1530 1538 10.1016/j.arthro.2010.04.065 20888170

[B71] Kocher MS Saxon HS Hovis WD Hawkins RJ Management and complications of anterior cruciate ligament injuries in skeletally immature patients: survey of the Herodicus Society and The ACL Study Group J Pediatr Orthop 2002 22 452 457 12131440

[B72] Barber FA Anterior cruciate ligament reconstruction in the skeletally immature high-performance athlete: what to do and when to do it? Arthroscopy 2000 16 391 392 10.1016/S0749-8063(00)90084-7 10802477

[B73] Lovell MR Fazio V Concussion management in the child and adolescent athlete Curr Sports Med Rep 2008 7 12 15 10.1097/01.CSMR.0000308671.45558.e2 18296938

[B74] Iverson GL Brooks BL Collins MW Lovell MR Tracking neuropsychological recovery following concussion in sport Brain Inj 2006 20 245 252 10.1080/02699050500487910 16537266

[B75] Kirkwood MW Yeates KO Taylor HG Randolph C McCrea M Anderson VA Management of pediatric mild traumatic brain injury: A neuropsychological review from injury through recovery Clin Neuropsychol 2008 22 769 800 10.1080/13854040701543700 17896204 PMC2847840

[B76] Sim A Terryberry-Spohr L Wilson KR Prolonged recovery of memory functioning after mild traumatic brain injury in adolescent athletes J Neurosurg 2008 108 511 516 10.3171/JNS/2008/108/3/0511 18312098

[B77] Fazio VC Lovell MR Pardini JE Collins MW The relation between post concussion symptoms and neurocognitive performance in concussed athletes Neuro Rehabilitation 2007 22 207 216 17917171

[B78] Kirkwood MW Yeates KO Wilson PE Pediatric sport-related concussion: A review of the clinical management of an oft-neglected population Pediatr 2006 117 1359 1371 10.1542/peds.2005-0994 16585334

[B79] McCrory P Collie A Anderson V Davis G Can we manage sport-related concussion in children the same as in adults? Br J Sports Med 2004 38 516 519 10.1136/bjsm.2004.014811 15388528 PMC1724912

[B80] Purcell L Canadian Paediatric Society, Healthy Active Living and Sport Medicine Committee Sport-related concussion: Evaluation and management Paediatr Child Health 2014 19 153 158 24665227 10.1093/pch/19.3.153 PMC3959977

[B81] Halstead ME Walter KD The American Academy of Pediatrics, Council on Sports Medicine and Fitness Clinical Report – Sport-related concussion in children and adolescents Pediatr 2010 126 597 611 10.1542/peds.2010-2005 20805152

[B82] Moser RS Glatts C Schatz P Efficacy of immediate and delayed cognitive and physical rest for treatment of sports-related concussion J Pediatr 2012 161 922 926 10.1016/j.jpeds.2012.04.012 22622050

[B83] Davis GA Purcell LK The evaluation and management of acute concussion differs in young children Brit J Sports Med 2013 48 2 98 101 23613516 10.1136/bjsports-2012-092132

[B84] Sady MD Vaughan CG Bioia GA School and the concussed youth: Recommendations for concussion education and management Phys Med Rehabil Clin N Am 2011 22 701 719 10.1016/j.pmr.2011.08.008 22050944 PMC3208828

[B85] McGrath N Supporting the student-athlete’s return to the classroom after sport-related concussion J Athl Train 2010 45 492 498 10.4085/1062-6050-45.5.492 20831397 PMC2938323

[B86] Centers for Disease Control and Prevention Heads up to schools: Know your concussion ABC’s 2010 http://www.cdc.gov/concussion/HeadsUp/high_school.html accessed 11 June 2014

[B87] Purcell L What are the most appropriate return-to-play guidelines for concussed child athletes? Br J Sports Med 2009 43 Suppl 1 i51 i55 19433426 10.1136/bjsm.2009.058214

[B88] Chrisman SP Schiff MA Chung SK Herring SA Rivera F Implementation of concussion legislation and extent of concussion education for athletes, parents, and coaches in Washington State Am J Sports Meddoi:10.1177/0363546513519073 10.1177/0363546513519073 24510067

[B89] Ontario Ministry of Education Policy/Program Memorandum No. 158: School Board Policies on Concussion Ontario, Canada http://www.edu.gov.on.ca/extra/eng/ppm/158.pdf *(Accessed on 27 May 2014)*

[B90] Ogden JA Skeletal injury in the child 2000 New York: Springer-Verlag

[B91] Bible JE Smith BG Ankle fractures in children and adolescent Techn Orthop 2009 24 211 219 10.1097/BTO.0b013e3181b58eee

[B92] Peterson HE Epiphyseal growth plate fractures 2007 Berlin: Spring-Verlag

[B93] American Academy of Orthopedic Physicians Growth Plate Injuries http://orthoinfo.aaos.org/topic.cfm?topic=A00040

[B94] National Institute of Arthritis and Musculoskeletal and Skin Diseases (NIAMS) Growth Plate Injuries: Questions and Answers about Growth Plate Injuries 2011 http://www.niams.nih.gov/Health_info/Growth_Plate_Injuries/default.asp#1

[B95] Mehlman CT Koepplinger ME Growth plate (physeal) fractures Medscape http://emedicine.medscape.com/article/1260663-overview#showall

[B96] Cutler L Molloy A Dhukuram V Bass A Do CT scans aid assessment of distal tibial physeal fractures? J Bone Joint Surg Br 2004 86 239 243 10.1302/0301-620X.86B2.13624 15046440

[B97] Boutis K Narayanan UG Dong FF Mackenzie H Yan H Chew D Babyn P Magnetic resonance imaging of clinically suspected Salter-Harris I fracture of the distal fibula Injury 2010 41 852 856 10.1016/j.injury.2010.04.015 20494352

[B98] Salter RB Harris WR Injuries involving the epiphyseal plate J Bone Jt Surg (USA) 1963 45 587 622

[B99] Barmeda A Gaynor T Mubarek SJ Premature closure following distal tibia physeal fractures J Pediatr Orthop 2003 33 733 739 10.1097/00004694-200311000-00010 14581776

[B100] Lee SH Lee DH Baek JR Proximal humerus Salter type III physeal injury with posterior dislocation Arch Orthop Trauma Surg 2007 127 143 146 10.1007/s00402-006-0135-4 17165038

[B101] DiFiori J Caine D Malina R Wrist pain, distal radial growth plate injury, and ulnar variance in the young gymnast Am J Sports Med 2006 34 840 849 16493174 10.1177/0363546505284848

[B102] Jaramillo D Laor T Zaleske DJ Indirect trauma to the growth plate: results of MR imaging after episphyseal and metaphyseal injury in rabbits Radiology 1993 187 171 178 8451408 10.1148/radiology.187.1.8451408

[B103] Dwek JR Cardoso F Chung CR MR imaging of overuse injuries in the skeletally immature gymnast: spectrum of soft-tissue and osseous lesions in the hand and wrist Pediatr Radiol 2009 39 1310 1316 10.1007/s00247-009-1428-x 19847413 PMC2776148

[B104] Fabricant PD Jones KJ Delos D Cordasco FA Marx RG Pearle AD Warren RF Green DW Reconstruction of the anterior cruciate ligament in the skeletally immature athlete: a review of current concepts: AAOP exhibit selection J Bone Joint Surg Am 2013 e28 1 13 doi:10.2106/JBJS.L.00772 10.2106/JBJS.L.00772 23467876

[B105] Hewett TE Lindenfeld TN Riccobene JV Noyes FR The effect of neuromuscular training on the incidence of knee injury in female athletes: A prospective study Am J Sports Med 1999 27 699 706 10569353 10.1177/03635465990270060301

[B106] Myer GD Brunner HI Melson PG Paterno MY Ford KR Hewett TE Specialized neuromuscular training to improve neuromuscular function and biomechanics in a patient with quiescent juvenile rheumatoid arthritis Phys Ther 2005 85 791 802 16048426

[B107] Myer GD Ford KR Barber Foss KD Liu C Nick TG Hewett TE The relationship of hamstrings and quadriceps strength to anterior cruciate ligament injury in female athletes Clin J Sport Med 2009 19 3 8 10.1097/JSM.0b013e318190bddb 19124976 PMC9928500

[B108] Noyes FR Barber-Westin SD Neuromuscular retraining intervention programs: do they reduce noncontact anterior cruciate ligament injury rates in adolescent females? Arthroscop 2014 30 245 255 10.1016/j.arthro.2013.10.009 24388450

[B109] Ladenhauf HN Graziano J Robert G Marx RG Anterior cruciate ligament prevention strategies: are they effective in young athletes – current concepts and review of literature Curr Opin Pediatr 2013 25 64 71 10.1097/MOP.0b013e32835ad208 23274428

[B110] Schiff M Caine D O’Halleron R Injury prevention in sports Am J Lifestyle Med 2010 4 42 64 10.1177/1559827609348446

[B111] Emery CA Kang J Shrier I Goulet C Hagel BE Benson BW Nettel-Aguirre JR McAllister JR Risk of injury associated with body checking among Youth Ice Hockey Players JAMA 2010 303 2265 doi: 10.1001/jama.2010.755 10.1001/jama.2010.755 20530780

[B112] McCrea M Hammeke T Olsen G Leo P Guskiewicz K Unreported concussion in high school football players: Implication for injury prevention Clin J Sport Med 2004 14 13 17 10.1097/00042752-200401000-00003 14712161

[B113] Valovich McLeod TC Schwartz CD Bay RC Sport-related concussion misunderstanding among youth coaches Clin J Sport Med 2007 17 140 142 10.1097/JSM.0b013e31803212ae 17414483

[B114] Register-Mihalik JK Linnan LA Marshall SW Valovich McLeod TC Mueller FO Guskiewicz KM Using theory to understand high school aged athletes’ intentions to report sport-related concussion: implications for concussion education initiatives Brain Inj 2013 27 878 886 10.3109/02699052.2013.775508 23789865

